# Coping strategies, satisfaction with life, and quality of life in Crohn’s disease: A gender perspective using structural equation modeling analysis

**DOI:** 10.1371/journal.pone.0172779

**Published:** 2017-02-28

**Authors:** O. Sarid, V. Slonim-Nevo, A. Pereg, M. Friger, R. Sergienko, D. Schwartz, D. Greenberg, I. Shahar, E. Chernin, H. Vardi, L. Eidelman, A. Segal, G. Ben-Yakov, N. Gaspar, D. Munteanu, A. Rozental, A. Mushkalo, V. Dizengof, N. Abu-Freha, A. Fich, S. Odes, on behalf of the Israeli IBD Research Nucleus (IIRN)

**Affiliations:** 1 Spitzer Department of Social Work, Ben-Gurion University of the Negev, Beer-Sheva, Israel; 2 Department of Public Health, Faculty of Health Sciences, Ben-Gurion University of the Negev, Beer-Sheva, Israel; 3 Department of Gastroenterology and Hepatology, Soroka Medical Center, Beer-Sheva, Israel; 4 Department of Health Systems Management, Faculty of Health Sciences, Ben-Gurion University of the Negev, Beer-Sheva, Israel; 5 Faculty of Health Sciences, Ben-Gurion University of the Negev, Beer-Sheva, Israel; University Hospital Llandough, UNITED KINGDOM

## Abstract

**Objective:**

To identify coping strategies and socio-demographics impacting satisfaction with life and quality of life in Crohn’s disease (CD).

**Methods:**

402 patients completed the Patient Harvey-Bradshaw Index, Brief COPE Inventory, Satisfaction with Life Scale (SWLS), Short Inflammatory Bowel Disease Questionnaire (SIBDQ). We performed structural equation modeling (SEM) of mediators of quality of life and satisfaction with life.

**Results:**

The cohort comprised: men 39.3%, women 60.1%; P-HBI 4.75 and 5.74 (p = 0.01). In inactive CD (P-HBI≤4), both genders had SWLS score 23.8; men had SIBDQ score 57.4, women 52.6 (p = 0.001); women reported more use of emotion-focused, problem-focused and dysfunctional coping than men. In active CD, SWLS and SIBDQ scores were reduced, without gender differences; men and women used coping strategies equally. A SEM model (all patients) had a very good fit (X^2^_(6)_ = 6.68, p = 0.351, X^2^/df = 1.114, SRMR = 0.045, RMSEA = 0.023, CFI = 0.965). In direct paths, economic status impacted SWLS (β = 0.39) and SIBDQ (β = 0.12), number of children impacted SWLS (β = 0.10), emotion-focused coping impacted SWLS (β = 0.11), dysfunctional coping impacted SWLS (β = –0.25). In an indirect path, economic status impacted dysfunctional coping (β = –0.26), dysfunctional coping impacted SIBDQ (β = –0.36). A model split by gender and disease activity showed that in active CD economic status impacted SIBDQ in men (β = 0.43) more than women (β = 0.26); emotional coping impacted SWLS in women (β = 0.36) more than men (β = 0.14).

**Conclusions:**

Gender differences in coping and the impacts of economic status and emotion-focused coping vary with activity of CD. Psychological treatment in the clinic setting might improve satisfaction with life and quality of life in CD patients.

## Introduction

Crohn's disease (CD) is a chronic illness with onset usually in young adulthood and a fluctuating, unpredictable clinical course characterized by exacerbations and remissions.[1] Symptoms include abdominal pain, diarrhea, weight loss, presence of an abdominal mass, fever, malaise, and in many patients also arthralgia and other extra-abdominal complications. There is a need for continuous therapy with pharmaceutical and biologic medications, nutritional therapy, and recourse to surgery in a significant number of patients. The disease is characterized by absence from work with consequent loss of income, and a considerable financial outlay.[2]

Well-being in patients with CD refers to both their medical and socio-psychological status. The Short Inflammatory Bowel Disease Questionnaire (SIBDQ) is a specific health-related quality of life instrument used very often in studies of CD.[3,4] The SIBDQ is objective and measures the impact of CD on physical, social and emotional domains in a broad range of problems encountered by the patient. The SIBDQ is easily administered as a self-complete tool. By comparison, the Satisfaction With Life Scale (SWLS) is an instrument that measures an individual's overall personal, subjective, all-encompassing judgment of his global life satisfaction.[5] It comprises 5 general statements. The SWLS has been used in healthy persons and in a variety of illnesses, but not, to our knowledge, in CD patients.

Coping with disease also affects patient well-being. It is defined as a conscious, intellectual and behavioral process whereby people make adjustments in order to handle the disease, and continue to function in the social setting.[6] The Ways of Coping scale [7] successfully designated 52 different coping strategies into three strategies. Problem-focused coping strategies involve defining a problem, and then generating solutions. Emotion-focused coping strategies seek to reduce emotional distress associated with a particular situation through trying to change one's feelings about it. Dysfunctional coping is unhelpful, maladaptive, and ineffective in helping the individual manage stress; it includes activities like denial and the use of alcohol and drugs. The Brief COPE (Brief Coping Operations Preference Enquiry) version [8] consisted of 28 items arranged into 14 sub-scales grouped into problem-focused, emotion-focused and dysfunctional coping strategies, and was more useful in clinical research. The Brief COPE has both reliability and validity, with good internal consistency for all three coping strategies.[9] Coping strategies in CD patients however are still poorly understood. Part of this confusion results from the use of multiple coping instruments and sub-scales with varying definitions of the three coping strategies [10].

Gender medicine is receiving increased attention as an important factor affecting the course and management of chronic diseases. Social and cultural conditions yield different experiences for men and women. Women generally experience more stress in their lives than men, likely related to their different gender social role stereotypes.[11] In Israel, as in other westernized societies, the social role of women is still directed towards care-taking and the family, whereas men are expected to be the financial supporter of the family. This affects how the genders handle chronic disease.

In summary, gender roles, coping strategies, demography and disease activity are all interrelated in their effects on patient well-being. We carried out a concerted investigation of these relationships, in two stages. Firstly, we characterized the quality of life and satisfaction with life in a large CD cohort, and their use of emotion-focused, problem-focused and dysfunctional coping strategies. Secondly, we determined the impact of demography and coping strategies as mediators of satisfaction with life and quality of life, using structural equation modeling, while accounting for gender and disease activity.

## Material and methods

The study was approved by the Ethics Committee of each hospital, and patients signed an informed consent form. Patients completing the questionnaires electronically did not supply any identifying information or an e-mail address, and were deemed to have assented to participate in the study. All data were treated anonymously.

### Study population and setting

Adult (age ≥18 years) patients with CD were studied using self-administered socio-psychological questionnaires, available in paper form, or electronically. Patients were eligible to participate irrespective of duration or severity of illness, medications and surgery. Patients were recruited consecutively by two concurrent methods. Patients with a confirmed diagnosis of CD [12] were recruited when presenting for follow-up, or for acute (non-hospitalized) care, at the Out-Patient Gastroenterology Departments of five participating university-affiliated tertiary-care public hospitals distributed throughout Israel (Rambam Health Campus, Tel-Aviv Medical Center, Soroka Medical Center, Shaare Zedek Medical Center, Tel-Hashomer Medical Center). The patients completed paper questionnaires. Additionally, patients visiting the website of The Israel Foundation for Crohn's Disease and Ulcerative Colitis completed the same questionnaire on-line. The time period of data collection in this cross-sectional study was July 2013 through June 2015.

Patients provided information about gender, age, education, marital and family status, religion, economic status (self-rated on a scale of 1–5, where 1 = very poor and 5 = very rich), and place of birth. Patients recorded the duration of CD, medical treatments, and hospitalizations and surgery for CD. Data about co-morbidities were obtained from subjects recruited at the hospitals.

### Patient Harvey-Bradshaw Index (P-HBI)

The Harvey-Bradshaw Index [13] comprises 5 questions about disease activity and extra-intestinal manifestations in the past 24 hours, and yields a disease activity score. Patients with inactive disease have a score ≤4. The HBI has a question about the presence of an abdominal mass that must be completed by a physician. By omitting this question, Evertsz et al. have published the Patient Harvey-Bradshaw Index (P-HBI).[14] The P-HBI was used as a self-complete questionnaire.

### Brief cope

This measure [8,9] comprises 28 items; each item is rated on a 4 degree scale (1 = I do not do it at all, 4 = I do it very much). Items are grouped to yield 14 coping subscales that are grouped into 3 strategies: emotion-focused (emotional support use, positive reframing, humor, acceptance, religion), problem-focused (active coping, instrumental support use, planning), and dysfunctional coping (self-distraction, denial, substance use, behavioral disengagement, venting, self-blame). Cronbach's alpha of these sub-scales was 0.72, 0.85 and 0.74, respectively. The Brief COPE presents the present condition of the subject and was valid among Israeli persons.[15]

### Satisfaction with Life Scale (SWLS)

This instrument [5] measures the individual’s level of satisfaction with life at that moment in time. It includes five questions (q): "q1, my life is close to ideal; q2, conditions of my life are excellent; q3, I am satisfied with my life; q4, I have gotten the important things I want in life; q5, if I could live my life over, I would change almost nothing." Each question is rated on a 7-point scale (1 = not agree at all with the item, 7 = strongly agree). The possible range of this scale is from 1–7 per question. The summary score has a range of 5–35, with a higher value indicating a higher level of satisfaction with life. Cronbach's alpha was 0.89. The SWLS was valid among Israeli subjects.[16]

### Short Inflammatory Bowel Disease Questionnaire (SIBDQ)

This instrument measures disease-specific quality of life and has physical (systemic and bowel symptoms), social and emotional dimensions.[3,17] It consists of 10 items: each item refers to the last two weeks, and is rated on a 7 degree scale (1 = all the time, 7 = never). The total score is in the range 10–70. A higher value indicates a better quality of life. Cronbach's alpha was 0.9. The scale was valid among Israeli subjects.[18]

The SWLS, SIBDQ and Brief COPE are in the public domain, were used as published, and were not adapted to test the particular obstacles encountered by patients with CD. The scales are all in arbitrary units.

### Statistical analysis

All data collected were pooled in a single database. Patients eligible for analysis were required to have filled in all the questions of the P-HBI, SWLS, SIBDQ and Brief COPE Inventory. Data were analyzed using IBM SPSS Statistics 22 for Windows with AMOS module (Armonk, NY: IBM Corp.). P values < 0.05 determined statistical significance.

Descriptive statistics were used. Continuous variables are expressed as means and standard deviations for normally distributed variables, or medians and interquartile ranges for variables with non-normal distribution; categorical variables are expressed as frequencies and percentages. The socio-demographic variables were compared between men and women using Student’s t-test and chi-square tests. Psychological variables were compared using Student’s t-test. A correlation analysis of SWLS and SIBDQ was performed separately for men and women with the following variables: economic status, number of children, P-HBI and coping strategies.

To understand the relationships and interactions between the study variables, a path analysis was developed in the 402 patients of the cohort using AMOS.[19–22] A model including all the variables in this large cohort did not give good indices of fit. We therefore excluded all non-significant paths from the general model as presented here. The general model was drawn by placing demographic variables as predictors for SWLS and SIBDQ. Next, we added two residual covariance paths, the first between SWLS and SIBDQ since these are both the outcomes of the disease, and the second between the two coping strategies derived from the same COPE questionnaire. Our adding these two residual covariance paths was supported by the AMOS modification indices function. In an iterative process, we added pathways that improved the model's fit, and removed variables that did not add significantly to the model's fit. As a way to reduce measurement error in the model only summated scales were drawn. Due to the fact that coping sub-scales were extracted from a single questionnaire, the residual covariance between them was drawn. Given a postulated association between SIBDQ and SWLS, a covariance between these two was drawn as well. All models were estimated using the maximum likelihood estimation method. Since quality of the fit of SEM models is affected by sample size, multiple model fit indicators were assessed including *χ*^2^, the ratio of the *χ*^2^ to degrees of freedom (*χ*^2^/df), the comparative fit index (CFI), p value, a root-mean-square error of approximation (RMSEA), and the standardized root mean-square residual (SRMR). Since the critical value of each *χ*^2^ degree of freedom is 3.8, all values that showed less than this value for each degree of freedom were considered non-significant and therefore did not deviate from the saturated model. Values close to 0.95 for the CFI, close to 0.06 for the RMSEA, and close to 0.08 for the SRMR indicate a good fit of the data to the model.[22]

In the next stage, P-HBI was dichotomously divided into inactive (P-HBI≤4) and active (P-HBI≥5) state of disease. We constructed a four group model based on gender and P-HBI.

## Results

### Cohort characteristics

Of 579 patients recruited for the study, 169 were ineligible because their responses to the questionnaires were incomplete, and 8 individuals were under 18 years. The 177 excluded patients differed from the 402 patients included in the final study cohort, being slightly younger (mean age 36.4 ± 14.8 versus 38.7 ± 14.0 years, respectively, p = .024), and including more women (71.3% versus 60.7%, p = .017). P-HBI mean scores were not significantly different between excluded and included patients.

The final study cohort (Table 1) comprised 158 men and 244 women. Women in the cohort were somewhat older. There were no gender differences of education, family status, the number of children, birth-place, smoking, and religion. 96.3% of the patients were Jewish. Economic status was median 3 on a scale of 1 (poorest) to 5 (highest) in men and women. Women had more active disease than men, P-HBI 5.74 ± 4.71 and 4.75 ± 4.71, respectively (p = .01). Disease duration and the rate of hospitalization in the past year were similar in both genders. Psychological co-morbidity was reported by 5% of the patients recruited at the hospitals, was mild and did not require pharmacotherapy.

**Table 1:** Cohort characteristics (N = 402).

Variable	Men	Women	p-value
	Median (Min; Max) (IQR*)or Mean ± SD or N (%)	Median (Min; Max) (IQR*)or Mean ± SD or N (%)	
Number in cohort	158 (39.3%)	244 (60.7%)	
Age	36.5 ± 12.6	40.1 ± 14.7	.020
Education (study years)	14 (4;23) (12;16)	12 (5; 30) (12; 17)	.077
Number of children	1 (0; 9) (0; 3)	2 (0; 8) (0; 3)	.127
Economic status	3 (1; 5) (3; 4)	3 (1; 5) (3; 3)	.081
Past Smoker	78 (49.4%)	107 (43.9%)	.229
Current Smoker	38 (24.1%)	40 (16.4%)	.078
Working	116 (73.4%)	153 (62.7%)	.026
Family status			
Married or living together	91 (57.6%)	151 (61.9%)	.478
Single or divorced	65 (41.1%)	93 (38.1%)	
Religion			
Jewish	149 (94.3%)	238 (97.5%)	**
Muslim	7 (4.4%)	2 (0.8%)	
Christian	1 (0.6%)	1 (0.4%)	
Other	0 (0%)	3 (1.2%)	
Degree of religiosity			
Ultra-Orthodox	10(6.3%)	8 (3.3%)	.104
Religious	25 (15.8%)	27 (11.1%)	
Traditional	27 (17.1%)	60 (24.6%)	
Secular	96 (60.8%)	148 (60.7%)	
Place of birth			
Israel	87 (55.1%)	129 (52.9%)	.237
Asia-Africa	7 (4.4%)	15 (6.1%)	
Eastern Europe-FSU	16 (10.1%)	15 (6.1%)	
America-Western Europe	5 (3.2%)	15 (6.1%)	
Harvey-Bradshaw Index (P-HBI)	4.75 ± 4.71	5.74 ± 4.71	.010
Inactive disease (P-HBI ≤4)	94 (59.5%)	123 (50.4%)	.074
Active disease (P-HBI ≥5)	64 (40.5%)	121 (49.6%)	
Disease duration (years)	10.8 ± 8.7	11.4 ± 8.56	.318
Medications			
Mesalamine	51 (32.3%)	65 (26.6%)	.223
Immunomodulators	77 (48.7%)	122 (50.0%)	.804
Steroids	27 (17.1%)	55 (22.5%)	.185
Biological	72 (45.6%)	108 (44.3%)	.797
Surgery	68 (43.0%)	72 (29.5%)	.005
Hospitalization (last year)	35 (22.2%)	60 (24.6%)	.547

* Interquartile range

** The Pearson Chi-Square test is not applicable in this case.

Of the 402 patients, 289 (71.9%) completed the paper questionnaires and 113 patients (28.1%) completed the electronic questionnaires; their mean ages were similar, 39.5 ± 14.7 and 36.5 ± 11.9 years, and their P-HBI scores were 5.01 ± 4.47 and 6.21 ± 5.27, respectively (p = .026).

### Psychological measures

Results of the psychological measures by P-HBI and gender are given in Table 2. In individuals with inactive disease, the SIBDQ was higher in men compared to women, but the SWLS was similar in the genders. Women with inactive disease reported greater use of emotion-focused, problem-focused, and dysfunctional coping strategies than men. In persons with active disease there were no significant differences between men and women with respect to reported measures of SWLS, SIBDQ and use of coping strategies.

**Table 2:** Descriptive values of psychological measures by disease activity and gender.

Disease Activity*	Measures (range)	Men (N = 158)	Women (N = 244)	p-value
		Mean ± SD	Mean ± SD	
Inactive	SWLS (5–35)	23.8±6.4	23.8±6.7	.977
	SIBDQ (10–70)	57.4±9.8	52.6±10.7	.001
	Emotion-focused Coping (10–40)	23.2±5.4	24.9±5.9	.025
	Problem-focused Coping (6–24)	14.8±4.9	16.8±4.8	.002
	Dysfunctional Coping (12–48)	19.8±5.9	21.9±5.5	.007
Active	SWLS (5–35)	20.0±8.6	20.9±7.8	.487
	SIBDQ (10–70)	39.9±12.7	38.1±12.2	.345
	Emotion-focused Coping (10–40)	24.4±5.9	24.3±5.3	.892
	Problem-focused Coping (6–24)	15.4±4.0	16.2±4.4	.192
	Dysfunctional Coping (12–48)	23.2±5.3	23.4±4.9	.573

*by P-HBI

SWLS, Satisfaction with life scale; SIBDQ, Short inflammatory bowel disease questionnaire

### Correlation coefficient matrix

A correlation coefficient matrix of the demographic, medical and psychological characteristics of the cohort appears in Table 3. P-HBI was negatively correlated with economic status in both genders. Problem-focused coping strategies were positively correlated with emotion-focused coping strategies in men and women. Dysfunctional coping strategies were positively correlated with emotion-focused coping strategies and problem-focused coping strategies; these correlations were higher in men compared to women. SIBDQ correlated positively with economic status and negatively with P-HBI and dysfunctional coping strategies, but without gender differences. SIBDQ correlated negatively with emotion-focused coping strategies in men. The significant correlation of SWLS with economic status was higher in men than women. SWLS was correlated positively with emotion-focused coping strategies in women, and negatively with problem-focused coping strategies in men. SIBDQ was correlated with SWLS (p<0.01) in men and women. A further correlation coefficient matrix of the demographic, medical and psychological characteristics of the cohort, separated by disease activity (active or inactive) and by gender, appears in the Supplementary file (Table A in S1 File).

**Table 3:** Pearson correlation coefficients matrix of the demographic, medical and psychological characteristics of the cohort.

	Economic status		No. children		P-HBI		Emotion-focused Coping Strategies		Problem-focused Coping Strategies		Dysfunctional Coping Strategies		SIBDQ	
	F	M	F	M	F	M	F	M	F	M	F	M	F	M
**No. children**	.071	.121												
**P-HBI**	-.162*	-.340**	.169**	.105										
**Emotion-focused**	.103	-.173*	-.054	-.067	-.041	.080								
**Problem-focused**	.029	-.180*	-.199**	-.182*	-.058	.021	.605**	.563**						
**Dysfunctional**	-.238**	-.278**	-.065	.010	.172**	.230**	.264**	.510**	.278**	.538**				
**SIBDQ**	.306**	.444**	-.01	-.081	-.626**	-.696**	.049	-.181*	-.048	-.151	-.337**	-.471**		
**SWLS**	.392**	.548**	.215**	.113	-.241**	-.281**	.246**	-.093	.011	-.246**	-.214**	-.364**	.435**	.510**

*p≤0.05;

** p≤0.01

Correlations in bold squares indicate significant difference between the genders

Correlations between SWLS or SIBDQ with age, education and disease duration did not reach significance and therefore were not included.

### General model

**Fig 1:**
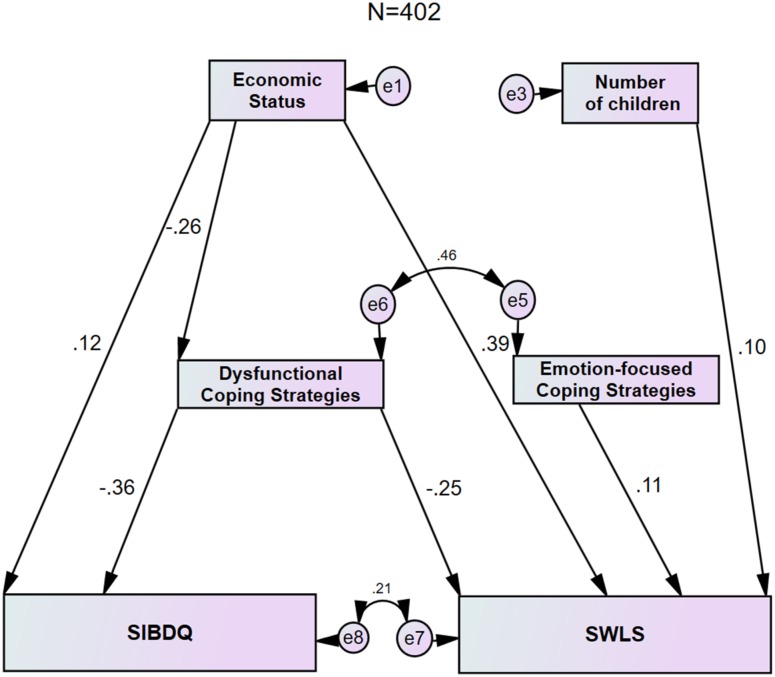
General model with path analysis showing the direct and indirect effects of economic status and number of children on SIBDQ and SWLS, with emotion-focused and dysfunctional coping strategies as mediators. Each of the coefficients in the model exceeds p≤0.05 significance.

The general model based on 402 patients (Fig 1, Table 4) gave a very good fit (*χ*^2^_(6)_ = 6.68, p = 0.351, *χ*^2^/df = 1.114, SRMR = 0.045, RMSEA = 0.023, CFI = 0.965), and presented several direct pathways by which SIBDQ and SWLS were predicted. The general model addresses 16% of SIBDQ and 26% of SWLS variances. Direct pathways linked the demographic variables of economic status with SIBDQ and SWLS, and number of children with SWLS. A better economic status led to a higher SIBDQ (standardized regression weight β = 0.12, p≤0.05) and better SWLS (β = 0.39, p≤0.001), whereas a higher number of children positively affected SWLS (β = 0.10, p≤0.05). Direct pathways linked coping strategies with SWLS and SIBDQ. A positive effect of emotion-focused coping strategies on SWLS was noted (β = 0.11, p≤0.05), and a negative effect of dysfunctional coping strategies on both SIBDQ (β = –0.36, p≤0.001) and SWLS (β = –0.25, p≤0.001) was observed. Thus, a greater use of dysfunctional coping was related to reduced SWLS and SIBDQ. Furthermore, economic status affected SIBDQ negatively in a non-direct pathway with dysfunctional coping strategies as mediator: economic status impacted dysfunctional coping (β = –0.26, p≤0.001) and dysfunctional coping affected SIBDQ (β = –0.36, p≤0.001). Of note, when problem-focused coping strategies was added as a mediator in the general model, no standardized regression weights of the path with SWLS or SIBDQ reached statistical significance, and the resultant model deviated significantly from the optimal model. To challenge the role of dysfunctional coping strategies as a mediator between economic status and SIBDQ, and the impact of dysfunctional coping strategies on SWLS, an alternative model was constructed where these mediating paths were set to zero. The alternative model (Table 4) showed a poor fit which deviated from the general model (*χ*^2^ difference 19.55, df = 1; p≤0.01, CFI < 0.9 threshold). Thus the general model presented a significantly better fit than the alternative model.

**Table 4:** Comparison of structural alternative model and path magnitudes between four groups.

	Fit measures						Model comparisons*	
	X^2^ (df)	p. model	X^2^/df	SRMR	RMSEA	CFI	X^2^ (df) difference	p. difference
**Comparing structural alternative model**								
General model, no constraints	6.68 (6)	0.351	1.114	0.045	0.023	0.965		
Alternative model, without dysfunctional coping as mediator	26.23 (7)	<0.01	3.74	0.103	0.113	0.862	19.55 (1)	<0.01
**Comparing paths magnitude between four groups**								
Four groups model, no constraints**	30.36 (24)	0.173	1.265	0.076	0.026	0.923		
Constraining five paths as follows: Dysfunctional Coping Strategies on SIBDQ Dysfunctional Coping Strategies on SWLS Economic status on Dysfunctional Coping Strategies Number of children on SWLS Economic status on SWLS	44.23 (39)	0.26	1.134	0.081	0.018	0.984	13.86 (15)	N.S
Constraining two paths as follows: Emotional cope on SWLS ES on SIBDQ	51.55 (30)	<0.05	1.72	0.075	0.042	0.869	21.19(6)	<0.01

*Model comparisons are conducted relative to the general model and four groups, respectively

**Cohort divided by gender and disease activity state

### Four groups model

**Fig 2:**
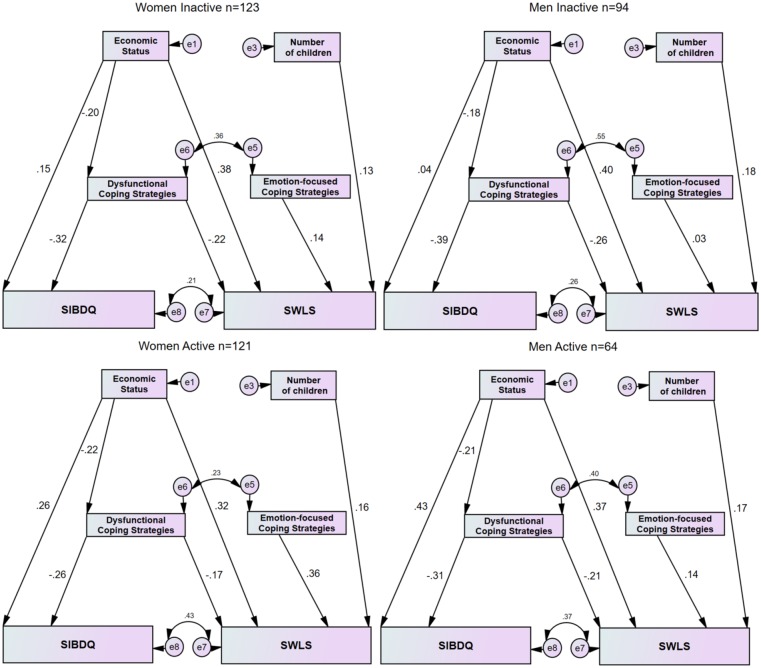
Four groups model path analysis, with the cohort split by gender (women, men) and disease activity (inactive disease, active disease). All β coefficients ≥ 0.15 reached p≤0.05 significance.

Following the univariate analysis that showed gender differences between the psychological variables according to disease activity (Table 2), we modified the general model into a four groups model based on gender and P-HBI. This model (Fig 2, Table 4) showed a very good fit (*χ*^2^_(24)_ = 30.36, p = 0.173, *χ*^2^/df = 1.26, SRMR = 0.076, RMSEA = 0.026, CFI = 0.923(, and illustrated the differences of magnitude of the paths as follows. In the inactive disease state there were small differences of the standardized regression weights between men and women with respect to the direct and indirect effects of economic status, number of children, dysfunctional and emotion-focused coping strategies on SIBDQ and SWLS. Yet, in the active disease state, the magnitude of two direct paths predicting SIBDQ and SWLS differed between men and women. Specifically, a higher magnitude of economic status on SIBDQ was observed in men compared to women (β = 0.43, p≤ 0.001 and β = 0.26, p≤ 0.001, respectively), whereas the magnitude of emotional coping strategies on SWLS was greater in women than men (β = 0.36, p≤ 0.001 and β = 0.14 p≤ 0.001 respectively).

In the model fitting process we examined whether the magnitudes of the paths in all 4 groups were equivalent. This was assessed by developing a model that sequentially constrained each of the paths (Table 4). While constraining all paths, other than economic status on SWLS and emotional coping strategies on SWLS, to be equal between all groups the model reached a very good fit (*χ*^2^_(39)_ = 44.23, p = 0.26, *χ*^2^/df = 1.134, SRMR = 0.081, RMSEA = 0.018, CFI = 0.984). Thus it was concluded that the 5 paths are equivalent in the four groups. Solely constraining the two paths economic status on SWLS and emotional coping strategies on SWLS yielded a non-appropriate model fit (*χ*^2^_(30)_ = 51.55, p≤0.05, *χ*^2^/df = 1.72, SRMR = 0.075, RMSEA = 0.042, CFI = 0.869). In other words, failing to fit the constrained model indicated nonequivalent path magnitudes in all 4 groups.

## Discussion

The present study examined the impact of socio-demographic variables and coping strategies on the quality of life and satisfaction with life in CD patients, with attention to differences between men and women, and regard for the activity of the disease. Our large CD cohort meets the criteria for making a reliable gender comparison. The cohort was recruited from all over the country, and included patients in all stages of disease activity. Demographic variables including economic status were similar in the genders. The excess of women over men and the somewhat higher disease activity score in women are typical of CD cohorts, as is the higher rate of surgery in men. A less favorable clinical course in women with CD, as compared with men, has been reported.[23]

This is the first study, to our knowledge, that defines the relationship of demographic factors and coping strategies with satisfaction with life and quality of life in CD patients using SEM analysis. SEM has a number of advantages over descriptive or multivariable regression analysis. It allows for the simultaneous evaluation of all the study variables and possible pathways. Our final model was derived by adding pathways based on AMOS modification indices, and presents those pathways that provided the best fit statistically. Thus, our final model gives the most exact statement of the interactions of the variables and as such improves on the correlations reported.

The path analysis showed that a higher economic status directly increased the SWLS and SIBDQ. This is in agreement with a study in a large healthy Australian cohort showing that household income was positively correlated with satisfaction with life measured by SWLS.[24] Persons able to maintain employment had a higher SWLS score than unemployed persons.[25] A review of socioeconomic factors in inflammatory bowel disease showed that SIBDQ varied directly with the level of income.[26] We found for the first time that the effect of economic status on SIBDQ was dependent on disease activity and gender. The greatest impact of economic status on SIBDQ was in men with active disease. A similar gender difference of economic status on quality of life was reported in a Swedish study in persons with the irritable bowel syndrome.[27] The high magnitude effect of economic status on SIBDQ among men with active CD in our cohort is likely explained by the fact that in the Israeli culture, as in other western cultures, men perceive their primary social role as providers, and thus view their economic status as a measure of success. This is also in accord with the constructionist gender theory of what constitutes appropriate behavior for men [28] and indicates that the inability of the sick male to fulfill his social role directly affects his psychological well-being.

The SEM model showed that number of children positively affected SWLS in a direct path which was unaffected by disease activity or gender. A likely explanation for this result is that Israel is a country with a pro-natal public policy, and childbirth is highly encouraged and appreciated.[29,30]

A recent comprehensive review detailed twenty-two coping instruments that were used in CD and ulcerative colitis.[8] Even papers employing the COPE or Brief COPE have used different combinations of sub-scales to make up emotion-focused, problem-focused and dysfunctional coping strategies. Comparison of these reports is difficult. In our study we showed that emotion-focused coping strategies affected SWLS by a direct path. Emotion-focused coping strategies in the Brief COPE include emotional support, positive reframing, humor, acceptance and religion [8,9] and intuitively would advance a feeling of well-being in patients. Emotion-focused coping is the commonest form of coping employed by persons with CD.[31] A lack of impact of emotion-focused coping on quality of life, as in our cohort, was reported previously.[20] Another study however showed that emotion-focused coping was associated with reduced quality of life.[32] In our study emotion-focused coping was used more frequently by women than men, confirming a previous report.[33] Moreover, the impact of emotion-focused coping in active CD was greater in women than in men; men actually demonstrated poor coping skills in our model. The great use of emotion-focused coping in women may be explained by their adherence to the traditional female roles of mother and family care-taker, despite their disease.[34] Of note, dysfunctional coping impacted directly on SWLS, and was a mediator between economic status and SIBDQ. All these effects were negative, irrespective of gender and disease activity. We found significant positive correlations between all three coping strategies in both genders, yet in the SEM analysis problem-focused coping was not retained. Problem-focused and emotion-focused coping were also positively correlated in a study of patients with a stoma, but in a final path model problem-focused coping was excluded.[20]

We found that quality of life was higher in men than women with inactive disease, but was reduced to an equally low level in both genders when disease was active. Women with CD have a lesser quality of life than men [33,34]. Quality of life was lower in active versus inactive CD.[35–38] Our study however is the first that examines quality of life by gender and disease activity using a SEM analysis. We also measured satisfaction with life in CD patients for the first time, using the SWLS. Although satisfaction with life was not included with patient-reported-outcomes in a recent review [39] our data suggest that it may serve as a useful additional measure of CD-patient well-being. While the SWLS score dropped in active CD, in inactive disease it was similar to the score reported in healthy persons [5]. The lack of gender difference in satisfaction with life, regardless of activity of disease, agrees with a recent study of satisfaction with life in healthy men and women.[40]

The major strength of our study is the use of advanced statistical modeling to define the predictors of quality of life and satisfaction with life, specifically by gender and activity of disease. Further strengths include using well-tested instruments, a coping instrument that distinguishes clearly the three types of coping strategies, and a large national cohort. Our study has some limitations. Inclusion of patients via the internet precluded the use of the Montreal classification, but the P-HBI satisfactorily reflected disease activity at the time when the questionnaires were completed. The unexplained variance in the models is large, and possibly relates to environmental variables and lack of health-promoting behavior like diet and exercise. A longitudinal study would indicate if the features of the model vary over time. The SWLS measure in CD needs more study. Further research into coping strategies in men with CD is required, since they exhibited little benefit from emotion-focused coping.

In conclusion, we show that coping strategies are different in men and women, and that the impacts of economic status and emotion-focused coping on patient well-being vary with activity of CD. This has important implications for physicians working in the clinical setting. All patients should be taught to identify and avoid dysfunctional coping strategies, and should be encouraged to use more emotion-focused coping. Men coming from a low economic background would constitute an especially vulnerable population and are first in the requirement of supportive psychological therapy. Indeed, social and psychological workers should play a greater role in all departments treating CD patients.

## Supporting information

S1 File Table A. Pearson correlation coefficients matrix of the demographic, medical and psychological characteristics of the cohort, separated by the state of disease and by gender. (DOCX)
