# Supplementary material for: Coping strategies, satisfaction with life, and quality of life in Crohn’s disease: A gender perspective using structural equation modeling analysis
Source: PLoS One. 2017 Feb 28;12(2):e0172779. doi: 10.1371/journal.pone.0172779 (PMC5330481; doi:10.1371/journal.pone.0172779)
Supplement: S1 File — (DOCX) [file pone.0172779.s001.docx]

**Table A**. **Pearson correlation coefficients matrix of the demographic, medical and psychological characteristics of the cohort, separated by the state of disease and by gender.**

|  | **Economic status** | | | | | | | | |
| --- | --- | --- | --- | --- | --- | --- | --- | --- | --- |
|  | All States of Disease | | | Active Disease | | | Disease Remission | | |
|  | All | F | M | All | F | M | All | F | M |
| Number of children | 0.091 | 0.072 | 0.121 | 0.123 | 0.143 | 0.094 | 0.114 | 0.040 | 0.196 |
| Emotion-focused | -0.018 | 0.105 | -.173^*^ | -0.027 | 0.085 | -0.189 | -0.002 | 0.110 | -0.112 |
| Problem-focused | -0.070 | 0.029 | -.180^*^ | 0.057 | 0.138 | -0.089 | -.176^**^ | -0.093 | -.232^*^ |
| Dysfunctional | -.266^**^ | -.240^**^ | -.279^**^ | -.187^*^ | -0.148 | -0.241 | -.266^**^ | -.281^**^ | -.208^*^ |
| SIBDQ | .381^**^ | .313^**^ | .457^**^ | .360^**^ | .241^**^ | .546^**^ | .231^**^ | .251^**^ | 0.147 |
| SWLS | .460^**^ | .397^**^ | .550^**^ | .397^**^ | .307^**^ | .529^**^ | .469^**^ | .450^**^ | .501^**^ |

*p≤0.05;** p≤0.01

|  | **Number of children** | | | | | | | | |
| --- | --- | --- | --- | --- | --- | --- | --- | --- | --- |
|  | All States of Disease | | | Active Disease | | | Disease Remission | | |
|  | All | F | M | All | F | M | All | F | M |
| Number of children | 0.091 | 0.072 | 0.121 | 0.123 | 0.143 | 0.094 | 0.114 | 0.040 | 0.196 |
| Emotion-focused | -0.058 | -0.057 | -0.067 | -0.002 | 0.053 | -0.083 | -0.124 | -.187^*^ | -0.063 |
| Problem-focused | -.185^**^ | -.203^**^ | -.182^*^ | -.161^*^ | -0.178 | -0.149 | -.218^**^ | -.232^*^ | -.217^*^ |
| Dysfunctional | -0.026 | -0.067 | 0.010 | 0.011 | 0.010 | 0.006 | -0.102 | -.211^*^ | -0.005 |
| SIBDQ | -0.048 | -0.010 | -0.083 | -0.033 | 0.036 | -0.131 | 0.038 | 0.092 | -0.011 |
| SWLS | .171^**^ | .216^**^ | 0.113 | .225^**^ | .323^**^ | 0.069 | .154^*^ | 0.121 | 0.192 |

*p≤0.05;** p≤0.01

|  | **Emotion-focused Coping** | | | | | | | | |
| --- | --- | --- | --- | --- | --- | --- | --- | --- | --- |
|  | All States of Disease | | | Active Disease | | | Disease Remission | | |
|  | All | F | M | All | F | M | All | F | M |
| Number of children | -0.018 | 0.105 | -.173^*^ | -0.027 | 0.085 | -0.189 | -0.002 | 0.110 | -0.112 |
| Emotion-focused | -0.058 | -0.057 | -0.067 | -0.002 | 0.053 | -0.083 | -0.124 | -.187^*^ | -0.063 |
| Problem-focused | .592^**^ | .605^**^ | .563^**^ | .529^**^ | .584^**^ | .440^**^ | .638^**^ | .619^**^ | .642^**^ |
| Dysfunctional | .375^**^ | .264^**^ | .510^**^ | .299^**^ | .207^*^ | .429^**^ | .441^**^ | .328^**^ | .557^**^ |
| SIBDQ | -0.057 | 0.057 | -.184^*^ | 0.004 | 0.029 | -0.032 | -0.117 | 0.044 | -.280^**^ |
| SWLS | .111^*^ | .246^**^ | -0.093 | .241^**^ | .370^**^ | 0.040 | -0.006 | 0.109 | -0.177 |

*p≤0.05;** p≤0.01

|  | **Problem-focused Coping** | | | | | | | | |
| --- | --- | --- | --- | --- | --- | --- | --- | --- | --- |
|  | All States of Disease | | | Active Disease | | | Disease Remission | | |
|  | All | F | M | All | F | M | All | F | M |
| Number of children | -0.070 | 0.029 | -.180^*^ | 0.057 | 0.138 | -0.089 | -.176^**^ | -0.093 | -.232^*^ |
| Emotion-focused | -.185^**^ | -.203^**^ | -.182^*^ | -.161^*^ | -0.178 | -0.149 | -.218^**^ | -.232^*^ | -.217^*^ |
| Problem-focused | .592^**^ | .605^**^ | .563^**^ | .529^**^ | .584^**^ | .440^**^ | .638^**^ | .619^**^ | .642^**^ |
| Dysfunctional | .401^**^ | .278^**^ | .538^**^ | .317^**^ | .211^*^ | .495^**^ | .473^**^ | .355^**^ | .571^**^ |
| SIBDQ | -.116^*^ | -0.048 | -.159^*^ | -0.126 | -0.105 | -0.147 | -.171^*^ | -0.093 | -0.189 |
| SWLS | -0.088 | 0.011 | -.246^**^ | -0.001 | 0.096 | -0.196 | -.175^**^ | -0.104 | -.282^**^ |

*p≤0.05;** p≤0.01

|  | **Dysfunctional coping** | | | | | | | | |
| --- | --- | --- | --- | --- | --- | --- | --- | --- | --- |
|  | All States of Disease | | | Active Disease | | | Disease Remission | | |
|  | All | F | M | All | F | M | All | F | M |
| Number of children | -.266^**^ | -.240^**^ | -.279^**^ | -.187^*^ | -0.148 | -0.241 | -.266^**^ | -.281^**^ | -.208^*^ |
| Emotion-focused | -0.026 | -0.067 | 0.010 | 0.011 | 0.010 | 0.006 | -0.102 | -.211^*^ | -0.005 |
| Problem-focused | .375^**^ | .264^**^ | .510^**^ | .299^**^ | .207^*^ | .429^**^ | .441^**^ | .328^**^ | .557^**^ |
| Dysfunctional | .401^**^ | .278^**^ | .538^**^ | .317^**^ | .211^*^ | .495^**^ | .473^**^ | .355^**^ | .571^**^ |
| SIBDQ | -.415^**^ | -.343^**^ | -.479^**^ | -.355^**^ | -.325^**^ | -.396^**^ | -.403^**^ | -.317^**^ | -.461^**^ |
| SWLS | -.275^**^ | -.214^**^ | -.364^**^ | -.177^*^ | -0.079 | -.322^**^ | -.313^**^ | -.311^**^ | -.330^**^ |

*p≤0.05;** p≤0.01

|  | **SIBDQ** | | | | | | | | |
| --- | --- | --- | --- | --- | --- | --- | --- | --- | --- |
|  | All States of Disease | | | Active Disease | | | Disease Remission | | |
|  | All | F | M | All | F | M | All | F | M |
| Economic status | .381^**^ | .313^**^ | .457^**^ | .360^**^ | .241^**^ | .546^**^ | .231^**^ | .251^**^ | 0.147 |
| Number of children | -0.048 | -0.010 | -0.083 | -0.033 | 0.036 | -0.131 | 0.038 | 0.092 | -0.011 |
| Emotion-focused | -0.057 | 0.057 | -.184^*^ | 0.004 | 0.029 | -0.032 | -0.117 | 0.044 | -.280^**^ |
| Problem-focused | -.116^*^ | -0.048 | -.159^*^ | -0.126 | -0.105 | -0.147 | -.171^*^ | -0.093 | -0.189 |
| Dysfunctional | -.415^**^ | -.343^**^ | -.479^**^ | -.355^**^ | -.325^**^ | -.396^**^ | -.403^**^ | -.317^**^ | -.461^**^ |
| SWLS | .469^**^ | .452^**^ | .514^**^ | .510^**^ | .483^**^ | .571^**^ | .352^**^ | .364^**^ | .351^**^ |

*p≤0.05;** p≤0.01

|  | **SWLS** | | | | | | | | |
| --- | --- | --- | --- | --- | --- | --- | --- | --- | --- |
|  | All States of Disease | | | Active Disease | | | Disease Remission | | |
|  | All | F | M | All | F | M | All | F | M |
| Economic status | .460^**^ | .397^**^ | .550^**^ | .397^**^ | .307^**^ | .529^**^ | .469^**^ | .450^**^ | .501^**^ |
| Number of children | .171^**^ | .216^**^ | 0.113 | .225^**^ | .323^**^ | 0.069 | .154^*^ | 0.121 | 0.192 |
| Emotion-focused | .111^*^ | .246^**^ | -0.093 | .241^**^ | .370^**^ | 0.040 | -0.006 | 0.109 | -0.177 |
| Problem-focused | -0.088 | 0.011 | -.246^**^ | -0.001 | 0.096 | -0.196 | -.175^**^ | -0.104 | -.282^**^ |
| Dysfunctional | -.275^**^ | -.214^**^ | -.364^**^ | -.177^*^ | -0.079 | -.322^**^ | -.313^**^ | -.311^**^ | -.330^**^ |
| SIBDQ | .469^**^ | .452^**^ | .514^**^ | .510^**^ | .483^**^ | .571^**^ | .352^**^ | .364^**^ | .351^**^ |

*p≤0.05;** p≤0.01

|  | **P-HBI** | | |
| --- | --- | --- | --- |
|  | All | F | M |
|  |  |  |  |
| Economic status | -.243^**^ | -.162^*^ | -.342^**^ |
| Number of children | .146^**^ | .172^**^ | 0.105 |
| Emotion-focused | 0.015 | -0.041 | 0.080 |
| Problem-focused | -0.010 | -0.058 | 0.021 |
| Dysfunctional | .207^**^ | .172^**^ | .230^**^ |
| SIBDQ | -.666^**^ | -.633^**^ | -.707^**^ |
| SWLS | -.255^**^ | -.241^**^ | -.281^**^ |

*p≤0.05;** p≤0.01
